# Genome-Wide and Locus Specific Alterations in *CDC73/HRPT2*-Mutated Parathyroid Tumors

**DOI:** 10.1371/journal.pone.0046325

**Published:** 2012-09-28

**Authors:** Luqman Sulaiman, Felix Haglund, Jamileh Hashemi, Takao Obara, Jörgen Nordenström, Catharina Larsson, C. Christofer Juhlin

**Affiliations:** 1 Department of Oncology-Pathology, Karolinska Institutet, Karolinska University Hospital, Stockholm, Sweden; 2 Department of Molecular Medicine and Surgery, Medical Genetics Unit, Karolinska Institutet, Karolinska University Hospital, Stockholm, Sweden; 3 Department of Endocrine Surgery, Tokyo Women's Medical University Hospital, Tokyo, Japan; 4 Department of Molecular Medicine and Surgery, Endocrine Surgery Unit, Karolinska Institutet, Karolinska University Hospital, Stockholm, Sweden; Ohio State University Medical Center, United States of America

## Abstract

Mutations in the hyperparathyroidism type 2 (*HRPT2/CDC73*) gene and alterations in the parafibromin protein have been established in the majority of parathyroid carcinomas and in subsets of parathyroid adenomas. While it is known that *CDC73*-mutated parathyroid tumors display specific gene expression changes compared to *CDC73* wild-type cases, the molecular cytogenetic profile in *CDC73*-mutated cases compared to unselected adenomas (with an expected very low frequency of *CDC73* mutations) remains unknown. For this purpose, nine parathyroid tumors with established *CDC73* gene inactivating mutations (three carcinomas, one atypical adenoma and five adenomas) were analyzed for copy number alterations and loss of heterozygosity using array-comparative genomic hybridization (a-CGH) and single nucleotide polymorphism (SNP) microarrays, respectively. Furthermore, *CDC73* gene promoter methylation levels were assessed using bisulfite Pyrosequencing. The panel included seven tumors with single mutation and three with double mutations of the *CDC73* gene. The carcinomas displayed copy number alterations in agreement with previous studies, whereas the *CDC73*-mutated adenomas did not display the same pattern of alterations at loci frequently deleted in unselected parathyroid tumors. Furthermore, gross losses of chromosomal material at 1p and 13 were significantly (*p* = 0.012) associated with parathyroid carcinomas as opposed to adenomas. Quantitative PCR-based copy number loss regarding *CDC73* was observed in three adenomas, while all the carcinomas were diploid or showed copy number gain for *CDC73 gene*. Hypermethylation of the *CDC73* gene promoter was not observed. Our data could suggest that *CDC73*-mutated parathyroid adenomas exhibit a partly unique cytogenetic profile in addition to that of carcinomas and unselected adenomas. Furthermore, *CDC73*-mutated carcinomas displayed losses at 1p and 13 which are not seen in *CDC73*-mutated adenomas, making these regions of interest for further studies regarding malignant properties in tumors from *CDC73*-mutated cases. However, due to the small sample size, validation of the results in a larger cohort is warranted.

## Introduction

Germ-line inactivating mutations of the hyperparathyroidism type 2 (*HRPT2/CDC73*) gene located in chromosomal region 1q31.2 underlie the hereditary hyperparathyroidism-jaw tumor (HPT-JT) syndrome associated with parathyroid tumors in addition to tumors of the jaws and uterus, as well as various kidney lesions [1], [2]. Constitutional *CDC73* gene mutations are also associated with a subset of families presenting with familial isolated hyperparathyroidism (FIHP) [3], [4]. Moreover, somatic *CDC73* gene mutations are found in the majority of sporadic parathyroid carcinomas [5], [6], [7], an entity which is over-represented in the HPT-JT syndrome. Surprisingly, a high incidence of constitutional *CDC73* mutations was demonstrated in cases with outwardly sporadic, non-familial diseases [5], [7]. *CDC73* gene mutations in parathyroid adenomas seem rare, but are reported, especially in cystic or atypical adenomas [1], [7], [8], [9], [10], [11].

The protein product of the *CDC73* gene, parafibromin, is an intricate protein localized and functionally linked to the nuclear, nucleolar as well as cytoplasmic compartments [12]. Parafibromin has previously been considered a tumor suppressor protein based on its ability to induce apoptosis, down-regulate cyclin D_1_ levels, inhibit G_1_ to S phase transition as well as to regulate gene expression of various growth factors [13], [14], [15]. In addition, parafibromin exhibits histone modulating properties, such as recruiting histone methyltransferases and processing histone mRNAs [16], [17], [18]. Moreover, studies have identified a number of oncogenic properties regarding parafibromin, including activation of the Wnt signaling pathway through beta-catenin binding and association to the human RNA Polymerase II-Associated Factor (hPAF) complex [16], [19], [20].

Total or partial loss of parafibromin expression is frequently detected in parathyroid carcinomas, however without precise correlation between mutation and protein function [9], [21], [22], [23], [24]. Other alterations of the *CDC73* locus such as DNA copy number alterations (CNAs) and DNA methylation are less well studied. Analyses of chromosomal gains or losses in parathyroid tumors have confirmed frequent deletions of chromosomal regions at 1p, 11q, 11p, 15q, 18q and 22q in adenomas and at 1p and 13q in carcinomas [25], [26], [27], [28], whereas gain of chromosomal material is frequent at chromosome 7, 13q and 20q for adenomas and 1q and 16p for carcinomas [26], [27]. The frequency of these CNAs varies, but nonetheless proposes that several candidate tumor suppressor genes (TSGs) and proto-oncogenes are sheltered within these regions. However, no studies have yet analyzed *CDC73*-mutated parathyroid tumors using concurrent genome-wide high-resolution array comparative genomic hybridization (a-CGH) or single nucleotide polymorphism (SNP) microarray-based loss of heterozygosity (LOH). *CDC73*- and *MEN1*-mutated cases generally cluster separately using gene expression profiling [29]. Based on this and the association between *CDC73* gene inactivation and malignant clinical behavior [6], we speculated that *CDC73*-mutated cases also could have a different molecular cytogenetic profile. Moreover, the mechanisms for *CDC73* gene inactivation are only partly understood. For these purposes, we studied nine parathyroid tumors with established *CDC73* gene mutations by a-CGH, SNP microarray, *CDC73* copy number analysis and *CDC73* promoter bisulfite Pyrosequencing.

## Materials and Methods

### Ethics statement

All samples were collected with oral informed consent. Oral informed consent is the current golden standard procedure at the Karolinska University Hospital, and the obtained consent is clearly documented in the patient's medical files in agreement with the Swedish Biobank law, thereby documenting the process. As of this, no written consent is needed. All patients have been informed that the extirpated tumor tissue will be collected in the biobank and might be subject to further molecular analyses. Studies of parathyroid tumors collected using oral informed consent have been specifically approved by the Karolinska University Hospital Ethics Committee and by the Karolinska Institute Research Ethics Committee, and these approvals include collection of tumor and normal parathyroid tissues from patients undergoing surgery for primary hyperparathyroidism using the method described above.

### Tissue samples

Nine fresh frozen parathyroid tumors with established *CDC73* gene mutations were collected from eight patients operated at the Karolinska University Hospital in Sweden and the Tokyo Women's Medical University Hospital in Japan during 1994–2006. The clinical information for each case is summarized in Table 1. Histopathological evaluation of all the tumors was carried out by a pathologist following the World Health Organization guidelines (DeLellis RA, Lloyd RV et al. 2004). Five tumors were diagnosed as parathyroid adenomas (T1–5a), one as an atypical adenoma with unknown malignant potential (T5b) and the remaining as parathyroid carcinomas (T6–T8). The T5a and T5b tumors were obtained from the same patient. In the carcinoma group, one case was a primary parathyroid carcinoma (T6) and one was a local recurrence of parathyroid carcinoma (T7); both T6 and T7 exhibited a subsequent medical history of lung metastases. The remaining case (T8) was lung metastases of parathyroid carcinoma. All parathyroid carcinomas exhibited histopathologically malignant properties. Cases T2, T4 and T6–8 had sporadic PHPT and T3 had FIHP as previously published [1], [5]. Case T1 is a member of a family with additional cases of PHPT without other known features of the HPT-JT syndrome, while the family of case T5 was lost for follow-up.

**Table 1:** Characteristics of the 9 parathyroid tumors.

				*CDC73/HRPT2* sequence*					
Case	Sex	Parathyroid Sample	Familial/Sporadic	Exon	Nucleotide	Protein	Effect	Level	*CDC73* copy number
T1	M	Adenoma	Familial	Ex1	IVS1+1 G>C		Splicing	Constitutional	2 copies
				Int12	IIVS12-109 T>G		Polymorphism		
		Blood		Ex1	IVS1+1 G>C		Splicing	Constitutional	
T2	M	Adenoma	Sporadic	Ex1	c. 126 del 24		Splicing	Somatic	1 copy
		Blood			wild-type				
T3	F	Adenoma	Familial	Ex1	c. 128 G>A	W43X	Stop	Somatic	1 copy
			(1q linked)						
		Blood			wild-type				
T4	M	Adenoma	Sporadic	Ex1	c. 53 del T		Frameshift	Somatic	1 copy
		Blood			wild-type				
T5a	M	Adenoma	n.a	Ex1 allele 1	c.71del 72-90	W30X	Frameshift, stop	Somatic	2 copies
					IVS2+31insCCTA		Polymorphism		
		Blood		Ex1 allele 2	c.128-IVS1+1 delG	W45X	Frameshift; alternative splice	Constitutional	
T5b		Atypical adenoma		Ex1	c.128-IVS1+1 delG		Frameshift; alternative splice	Constitutional	2 copies
T6	F	Carcinoma	Sporadic	Ex1	c. 82 del 4		Frameshift @28 to Stop @35	Somatic	4 copies
				Ex8	732delT		Frameshift @244 to Stop @35		
		Blood			wild-type				
T7	M	Carcinoma	Sporadic	Ex1	c. 70 G>T	E24X	Stop	Somatic	2 copies
				Ex8	c. 746 del T		Frameshift @249 to Stop@256		
		Blood			wild-type				
T8	M	Carcinoma	Sporadic	Ex2	c. 226 C>T	R76X	Stop	Somatic	3 copies
		Blood			wild-type				

M = Male, F = Female, Ex = Exon, Int = Intron, n.a = not available.

LOH =  Loss of heterozygocity, n.d. = not determined.

* CDC73/HRPT2 mutations were previously published for cases T2–T4, and T6–T8 (References [1], [5]).

The constitutional mutation in T5 was revised from a previous publication (Reference [30]).

Frozen tissue samples were acquired from three normocalcemic, non-tumorous parathyroid tissues (N1, N2 and N3) and used as references for the bisulfite Pyrosequencing and DNA copy number analysis.

As an overview, 8 out of 9 cases were analyzed for CNAs using array-comparative genomic hybridization (a-CGH) and 7 out of 9 for loss of heterozygosity using single nucleotide polymorphism (SNP) microarray. Furthermore, *CDC73* gene promoter methylation levels were assessed in 8 out of 9 tumors using bisulfite Pyrosequencing.

### DNA sequencing

The *CDC73* sequencing data for cases T2–T4 and T6–T8 has been reported elsewhere [5], [22], [30]. Germ-line *CDC73* sequences were available from cases T1 and T5a records related to earlier diagnostics at the Karolinska University Hospital department of Clinical Genetics and/or by sequencing of constitutional tissues. For cases T1 and T5a-b with unknown *CDC73* gene mutational status, DNA was extracted from the tumor samples using Invitrogen ChargeSwitch (Invitrogen, Carlsbad, CA, USA) following the manufacturer's protocol. DNA concentrations were measured by Nanodrop A100 (Thermo Scientific, Waltham, MA, USA) and quality verified by agarose gel electrophoresis. All exons were amplified by PCR using previously assessed primers and DyNAzyme DNA Polymerase (New England Biolabs, MA, USA). Nucleotide sequences were determined using BigDyeTerminator 3.1 with an automated sequence scanner and sequences were analyzed using SeqScape v2.5 (Applied Biosystems, Foster City, CA, USA). Mutations were confirmed with a reverse sequencing reaction.

### DNA TaqMan copy number assay

DNA copy number analysis was performed for the *CDC73* locus using TaqMan copy number analysis (Applied Biosystems, Foster City, CA, USA) according to the description in [9]. All samples including the three normal reference parathyroid DNAs (N1–3) were amplified in the same 96 well plate in triplicate, each containing the same *CDC73* assay (Hs01964898_cn) along with the endogenous control *RNaseP* (Hs_cn 4403326) used for normalization purpose and calibrated to normal reference parathyroid DNA (N1–3). The *CDC73* gene copy numbers were predicted using CopyCaller software V1.0 (Applied Biosystems, Foster City, CA, USA).

### Bisulfite Pyrosequencing

The methylation density of the *CDC73* gene promoter as well as the global methylation status were analyzed for all the tumors (except T1 due to lack of DNA) and the three reference parathyroid samples (N1–N3) using the Pyrosequencing method as previously described [31]. Briefly, samples were bisulfite treated using EpiTect Bisulfite conversion kit (Qiagen AB, Sweden) and specifically amplified using pyromark CpG assay (PM00002625) for *CDC73* promoter methylation or Long Interspersed Element 1 (*LINE-1*) (Qiagen AB, Sweden). The PCR products were then run and analyzed using the PyroMark Q24 machine and PyroMark Q24 software, respectively (Qiagen AB, Sweden).

### Genome-wide high-resolution array-CGH (a-CGH)

All the tumor samples (except T1) were available for a-CGH analysis. DNA copy number alterations (CNAs) were investigated using the human BAC 38K Array CGH platform produced at SCIBLU Genomics Centre at Lund University, Sweden (www.lu.se/sciblu).

The protocol for the experimental procedures and subsequent data analysis (www.lu.se/sciblu) followed was previously described in [9]. Briefly, tumor DNA and sex mismatched normal reference DNA (Promega, Madison, USA) were differentially labeled with Cy3 and Cy5 fluorescent dyes (PA53021, PA55021, GE HealthCare), respectively, using random priming by Bioprime labeling kit (Invitrogen Life Tehcnoloigies, Carlsbad, CA, USA). Labeled DNA was purified, mixed with Cot-1 DNA (Life Technologies, Inc., CA, USA) and dried in speed vacuum. The samples were then re-suspended in hybridization solution and hybridized onto pre-treated array glass slides in water bath at 37°C for 72 hours. The same batch of arrays and identical labeling and hybridization conditions were used for all samples. After hybridization, slides were washed, immediately dried and then scanned in an Axon Scanner 4000A (Axon Instruments, Burlingame, CA, USA).

### Array-CGH data analysis

After scanning, features intensities were extracted using GenePix Pro 6.0 software (Axon Instrument, CA, USA). The generated gene pix result (GPR) files were uploaded to the web-based a-CGH analytic tool Bio Array Software Environment version 2 (BASE2) (http://base.thep.lu.se/). After importing the data, background correction was performed by subtracting median foreground from median background of Cy3 and Cy5 intensities respectively. Data was filtered for flagged bad or null spots. The samples were then normalized using BASE 1 LOWES plugin [32] and transformed using smooth default parameters with 3 clones sliding window size.

CNAs were visualized using CGH plotter applying cut-offs values for log2 ratio of 0.25 for gains, −0.25 for losses, +1 for amplifications and −0.8 for homozygous losses. Whole genome frequency plots and individual chromosome profiles were generated using BEFC viewer plugin in BASE2. Sex chromosomes were excluded from the final analysis.

### 250K single nucleotide polymorphism (SNP) microarray

For loss of heterozygosity (LOH) analysis, all tumors were run on Affymetrix 250K genotyping chip (Affymetrix Inc., Santa Clara, CA, USA) at the Bioinformatics and Expression Analysis Core Facility (BEA) in Karolinska Institutet, Sweden. Each SNP array slide contained 25,000 SNPs distributed randomly throughout the genome. The experimental setup and the analysis were performed following the standard protocol and analysis workflow of the Affymetrix Gene Chip 250K Mapping array manual. In brief, 250 ng of total genomic DNA was enzymatically treated with the restriction enzyme *StyI* and the fragments were ligated and PCR amplified to reduce the complexity of the genomic DNA. The amplified product was then further fragmented, labeled and hybridized to the SNP array slides. Following hybridization, slides were washed, dried and scanned in a GeneChip scanner 3000 (Affymetrix).

### 250K SNP array analysis

Genotype files (CHP) were generated by the Genotype console (Affymetrix Inc. Santa Clara, CA, USA) using the Bayesian Robust Linear Model with Mahalanobis (BRLMM) algorithm. The CHP files were then imported into Partek Genomic Suite V6.5 software (Partek Incorporated. St. Louis, MO) using the Hidden Markov Model (HMM). The default Partek parameters were used for further LOH analysis including a heterozygosity call rate of 0.3 and the minimum number of consecutive probes required to a make call set as 10 probes. All samples were checked for quality and as both T5a and T1 did not pass the QC they were excluded from further analysis. Similar to a-CGH, sex chromosomes were excluded from the final analysis. Based on all the genomic alterations detected, unsupervised hierarchical clustering of all the *CDC73*-mutated tumors was performed using Euclidean algorithm with average linkage and zero distance using Partek Genome Suite 6.5 (Partek inc. MI, U.S.A).

### Statistical analyses

Fisher exact test was used to estimate any significant differences in the distribution of the CNAs between the adenoma and the carcinoma groups. Considering the sample size and at power of 80%, a *p* value of <0.05 was regarded as statistically significant. Statistical analyses were performed using SigmaPlot (SigmaPlot for windows v11.0, SYSTAT software inc, Hamburg, Germany).

## Results

### *CDC73* gene mutations

**Figure 1:**
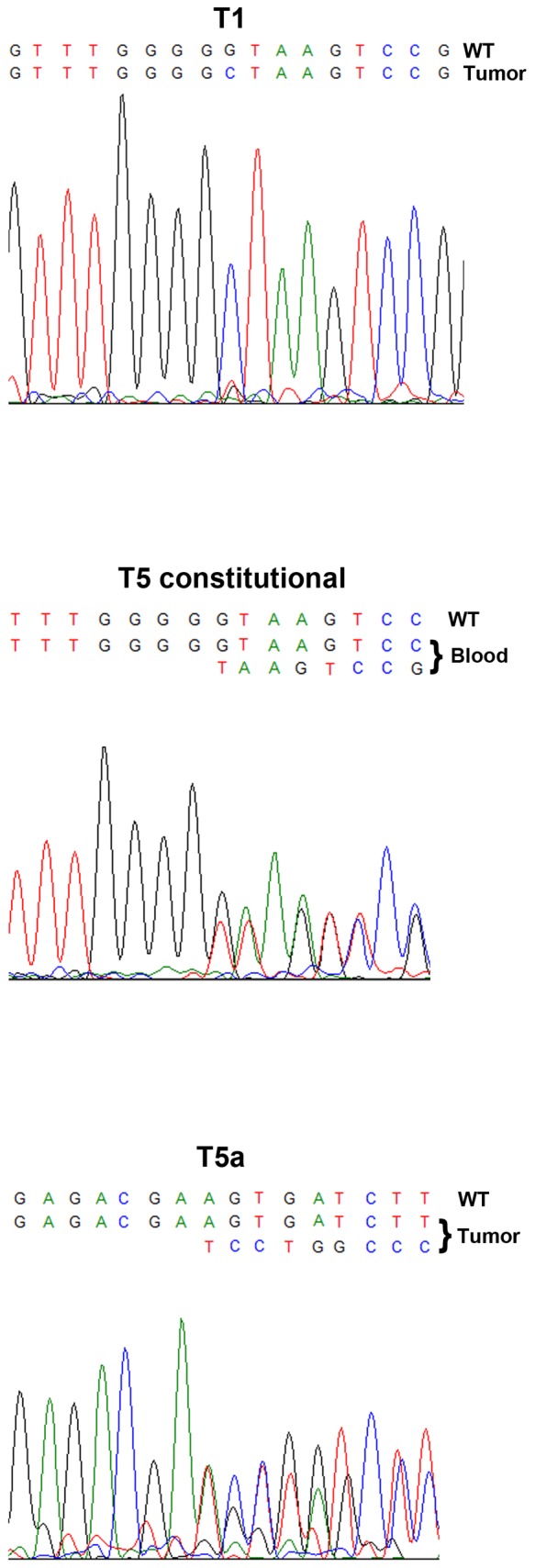
Sequencing chromatograms of *CDC73* mutations in parathyroid tumors: T1 (IVS1+1 G>C), T5 constitutional (c128-IVS1+1 delG) and T5a (c.71del 72–90). Wild-type (WT) sequences are indicated above the mutated sequences.

Details of the mutations for each case and their predicted effects are shown in Table 1. Chromatograms for the previously unreported cases are visualized in Figure 1. The three tumors from cases T1, and T5a-b were fully sequenced and four *CDC73* mutations were detected. The two patients presenting with T1 and T5a-b both carried constitutional *CDC73* mutations; IVS1+1 G>C in patient T1 and c.128-IVS1+1 delG in patient T5a-b. The same mutations were detected in the corresponding tumors. For T1, no additional mutation was identified in the tumor; however the sequencing chromatogram showed only one peak as compared to the blood sequencing. In T5a an additional somatic mutation c.71del 72–90 was detected. Since the constitutional and the somatic mutations were present in the same chromatogram, it was possible to distinguish that the respective mutation was present on different alleles. No additional mutations were found in T5b.

Overall, three tumors (T5a, T6, T7) displayed possible bi-allelic mutations and the remaining seven tumors demonstrated mono-allelic mutations. The mutations in T3–T8 are predicted to prematurely truncate parafibromin due to frameshift alterations or nonsense mutations. However, T1 and T2 are predicted to involve the first intron sequence and the first nucleotide of the consensus donor splice site of intron 1.

### *CDC73* gene copy number analysis

**Figure 2:**
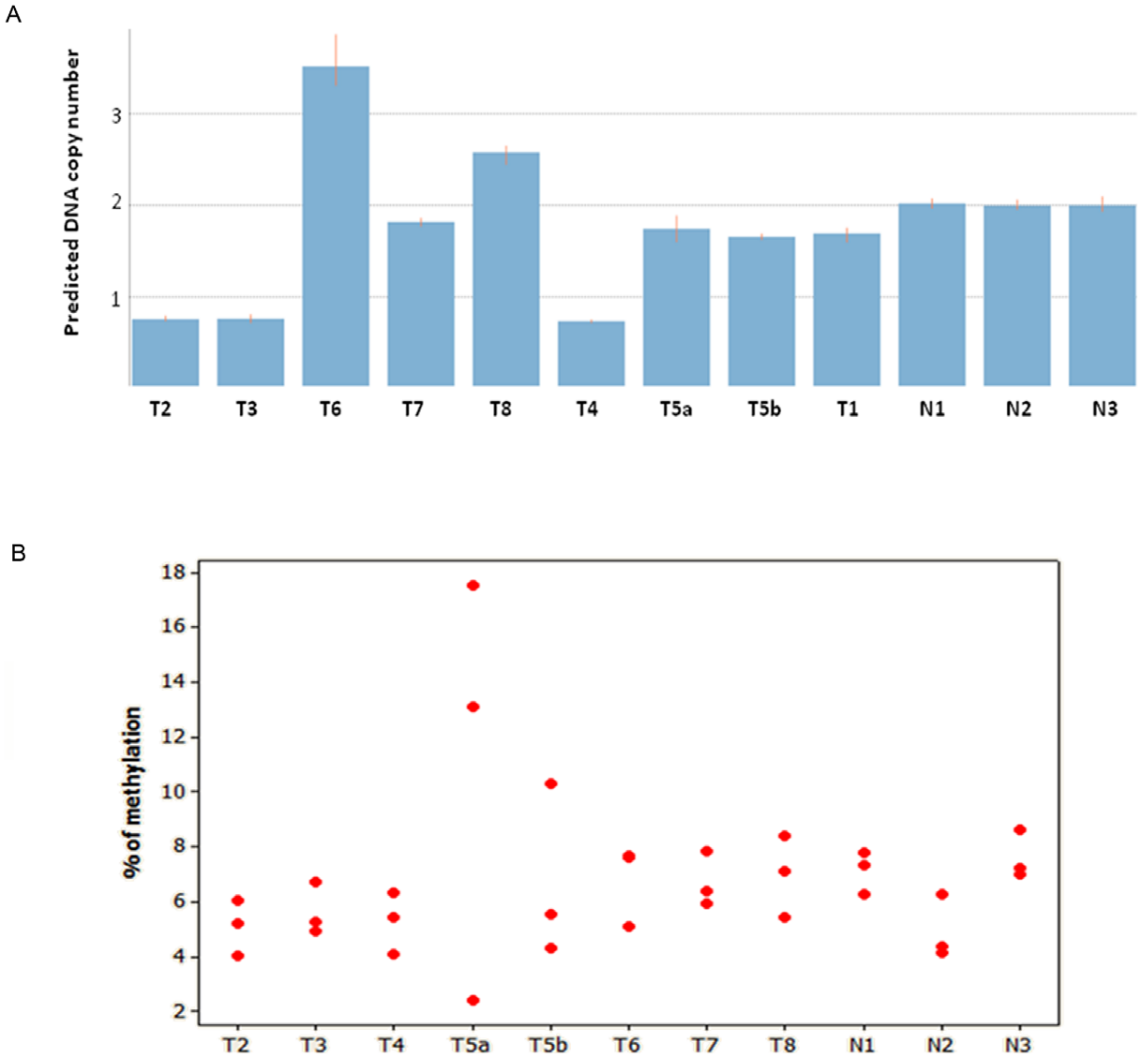
(A) Bar graph showing predicted DNA copy number of the *CDC73* gene using TaqMan DNA copy number analysis. Each bar represents one parathyroid sample and the height of the bar represents the predicted *CDC73* gene DNA copy number. T2, T3 and T4 had 1 copy of *CDC73* gene, while T6 and T8 had more than 2 copies. N1, N2 and N3 refer to the normal parathyroid samples used as calibrators. (B) Individual value plot illustrating the methylation density at the three analyzed CpG dinucleotides of the *HPRT2* promoter. Each red dot represents one CpG site in one parathyroid tumor numbered from T2–T8 along with the three normal references denoted as N1, N2 and N3.

Nine of the tumors (T1–T8) were analyzed for CNAs of the *CDC73* locus using TaqMan copy number analysis. Samples T2, T3 and T4 displayed only 1 copy while T6 and T8 had 4 and 3 copies, respectively. The remaining tumors (T1, T5a, T5b and T7) were diploid for the *CDC73* gene locus (Figure 2A).

### *CDC73* gene specific and global methylation analysis

Eight tumors (T2–T8) were investigated for CpG methylation density of the *CDC73* promoter and compared to the three normal references by Pyrosequencing technique. The reference samples showed a mean methylation density of 7% (5%–8%). Seven tumors showed similar findings with a mean methylation of 6% (5%–7%). For T5a, an increased methylation density was noted for 2/3 of the CpGs (13% and 18%) (Figure 2B). No alterations in the global methylation level were observed when compared to the reference samples and all had a *LINE-1* mean methylation level of about 70%.

### Overall a-CGH findings

**Figure 3:**
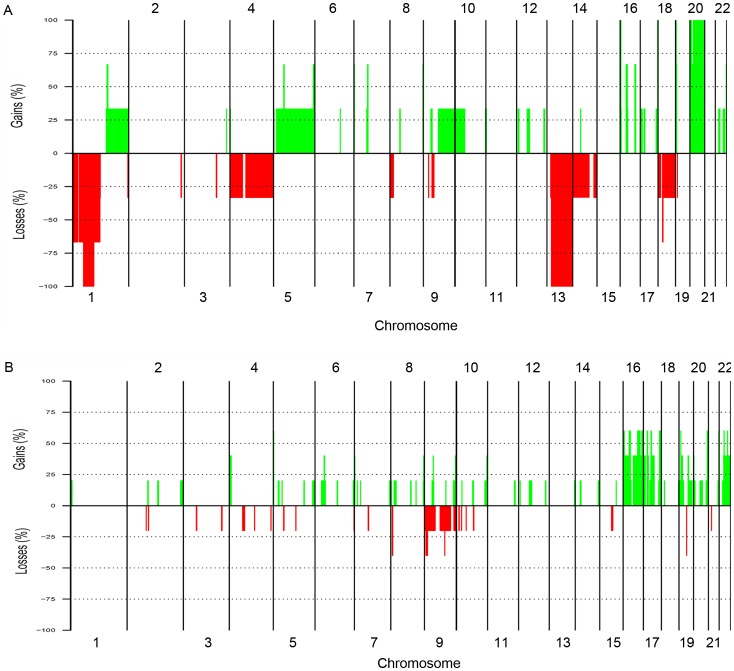
Whole genome frequency plot showing CNAs detected by a-CGH; (A) in the parathyroid carcinomas (T6–T8); and (B) in the parathyroid adenomas (T2–T5a/b) analyzed. Gains are illustrated in green and losses are shown in red. CNAs are arranged based on the chromosome order from 1 to 22. Sex chromosomes were excluded from the analysis.

Eight tumors (four adenomas, three carcinomas and one atypical adenoma) were screened for genome-wide DNA copy number alterations (CNAs) using a-CGH technique. All tumors displayed CNAs. Overall, gains were more common than losses on both large-scale and sub-chromosomal levels (Figure 3). Tumor T7 showed the largest total CNAs (>800 Mb), while T2 showed the smallest (<5.71 Mb). No amplifications or homozygous losses were detected. The common minimal recurrent CNAs observed in at least two tumors were losses of 1p21.2–13.3 (38%), 9p23–24.1 (25%) and 18q11.2 (25%) and gains of 1q21.3–23.1 (25%), 1q32.1 (25%), 5q31.1 (25%), 9p13.1–21.1 (25%), 11q12.2–13.2 (25%), 12q13.11–14.1 (25%), 16q22.1 (63%), 16p11.2 (63%), 17p11.2 (50%), 18p11.21 (25%), 20q11.21 (63%), 21q22.3 (38%), 22q11.23 (38%) and 22q13.1 (63%). All the common minimal recurrent CNAs detected are summarized in Table 2.

**Table 2:** Most common recurrent copy number alterations detected by a-CGH in CDC73-mutated parathyroid tumors (T2–T8) with corresponding region of LOH.

Cytoband	Start Clone	End Clone	Position (Mb)	Size (Mb)	No. Of Clones	Freq %	Tumors Affected	SNP-based LOH	Examples*
***Losses***									
1p21.2-13.3	RP11-721B18	RP11-483I13	102740298-108610118	5.9	75	38%	T6, T7, T5b	No	*RNPC3, VAV3*
9p23-24.1	RP11-403H13	RP11-382H24	6858099-13122077	6.2	73	25%	T4, T5b	No	*PTPRD*
18q22.1-22.2	RP11-26L13	RP11-49H23	63370817-65089302	1.7	22	25%	T6, T7	Yes	*TXNDC10*
***Gains***									
1q21.3-23.1	RP11-744J16	RP11-711O18	150155440-153665553	3.5	43	25%	T6, T8	Yes	*S100A5*
1q32.1	RP11-115O14	RP11-161C18	196402802-203224723	6.8	88	25%	T6, T5B	Yes	*ELK4*
5q31.1	RP11-21C10	RP11-158M10	133880078- 135502898	1.6	24	25%	T6, T7	Yes	*CAMLG, DDX46, CATSPER3, PITX1, TIFAB*
9p13.1-21.1	RP11-340D22	RP13-198D9	32618776-38734139	6	74	25%	T7, T5b	No	*FANCG, PAX5*
11q12.2-13.2	RP11-727C13	RP11-428E19	60661723-67118617	6.5	82	25%	T8, T5b	No	*MEN1*
12q13.11-14.1	RP11-204C20	RP11-571M6	46894554- 56496966	9.6	138	25%	T8, T5b	No	*CDK4, DDIT3 , HOXC11 and 13, NACA*
16q22.1	RP11-1150F18	RP11-6M2	64948284- 69264998	4.3	61	63%	T3, T5a, T5b, T6, T8	No	*CBFB, CDH1*
16p11.2	RP11-544O7	RP11-466D2	27824814- 31535714	3.7	49	63%	T3, T5a, T5b, T6, T8	No	*FUS*
17p11.2	RP11-537F12	RP11-34O10	16592363- 18318880	1.7	21	50%	T3, T5a, T5b, T8	No	*FLCN, MED9, MIR33B*
18p11.21	RP11-44O1	RP11-510L15	12610439- 13384576	0.8	11	25%	T7, T8	No	*PTPN2*
20q11.21	RP11-802B20	RP11-471K3	29454263-31300949	1.8	21	63%	T5a, T5b, T6, T7, T8	No	*ASXL1*
21q22.3	RP11-1007O14	RP11-726J13	44825264- 45558273	0.7	10	38%	T3, T5a, T5b	No	*PTTG1IP,*
22q11.23	RP11-164N13	RP11-698L6	21892457-22518106	0.6	4	38%	T2, T5a, T5b	No	*SMARCB1, BCR*
22q13.1	RP11-555L22	RP11-168F23	36717836- 37293012	0.6	4	63%	T5a, T5b, T6, T7, T8	No	*DDX17, DMC1*

* Genes written in bold are known cancer genes according to Cancer Gene Census, Welcome Trust, Sanger Institute.

### CNAs in parathyroid carcinomas

The three parathyroid carcinomas (T6, T7 and T8) displayed large-scale losses of chromosome 1p, loss of the entire chromosome 13 and gain of 20. In addition, T6 displayed gross loss of chromosome 14, while T7 had the most extensive aberrations with large-scale losses of chromosomes 4 and 18 and large-scale gains of chromosomes 5, 9, 10 and 22 (Figure 3A).

At the subchromosomal level, T8 shared a small region of gain at 1q21.3–23.1 with T6, which displayed gain of entire 1q arm. This region overlaps with few genes most importantly the S100 A5. Similarly, T8 also shared a 1.6 Mb region of gain at 5q31.1 with T7 which displayed entire chromosome 5 gain. This region contains several genes involved in calcium signaling and cellular growth such as *CAMLG*, *DDX46*, *CATSPER3* and *TIFAB*. None of the carcinomas exhibited loss on chromosome 11.

### CNAs in parathyroid adenomas and atypical adenoma

Overall, the adenomas (T2, T3, T4 and T5a) had the least extent of CNAs (Figure 3B). The adenoma T2 did not display any significant aberrations, while T4 displayed deletion of the entire chromosome 9 and shared a 6.2 Mb region of loss at 9p23–24.1 with the atypical adenoma case (T5b). Gain of the entire chromosome 16 was detected in T3 and T5a, which also displayed gross gain of entire chromosome 17 and 22, respectively. None of the adenomas showed any significant aberrations on chromosome 1, 11 or 13 (Figure 3B). The atypical adenoma (T5b) had extensive aberrations which included subchromosomal changes affecting almost all chromosomes along with large-scale gains on 1p, 16, 17, 19, 20 and 22. This case shared small regions of gains on chromosome 11 and 12 with the carcinoma case T8. This included gains of 11q12.2–13.2 and 12q13.11–14.1 which overlapped with many genes including *MEN1* and *CDK4*, respectively. Apart from loss at 1p21.2–13.3 shared with the carcinomas T6 and T7, no other significant deletions could be detected in the atypical adenoma case. Compared to the atypical adenoma (T5b), the adenoma T5a originating from the same patient had less extensive CNAs. However, they partly showed similar genomic profiles regarding gains on chromosome 16, 17p and 17qter, 20q11, 21q22 and 22.No losses spanning the *CDC73* gene locus at 1q31.2 were detected among the carcinomas or in the atypical adenoma case.

### Clustering of array CGH data

**Figure 4:**
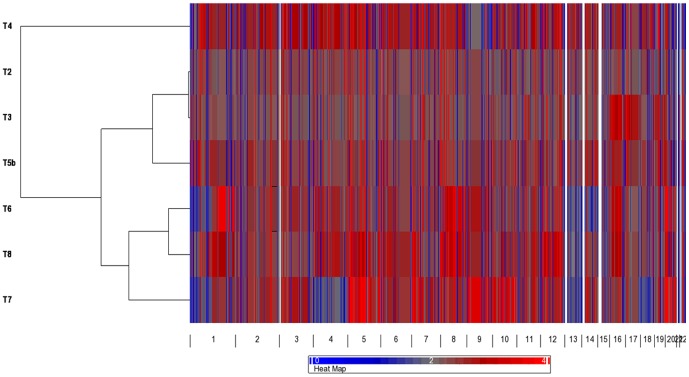
HeatMap representing unsupervised hierarchical whole genome clustering incorporating all the CNAs detected by a-CGH (with the exclusion of T1 and T5a). Three clusters were identified. One cluster grouped the carcinomas T6, T7, and T8, another cluster included T2, T3 and T5b, while T4 grouped separately. Grey refers to no changes, red to gain and blue to loss.

Unsupervised hierarchical clustering of the CNA data divided the eight tumors analyzed into three main clusters with the carcinomas (T6, T7 and T8) falling into one cluster, T2, T3 and T5b in another cluster, while T4 clustered separately (Figure 4).

### SNP-based detection of Loss of Heterozygsity (LOH)

**Figure 5:**
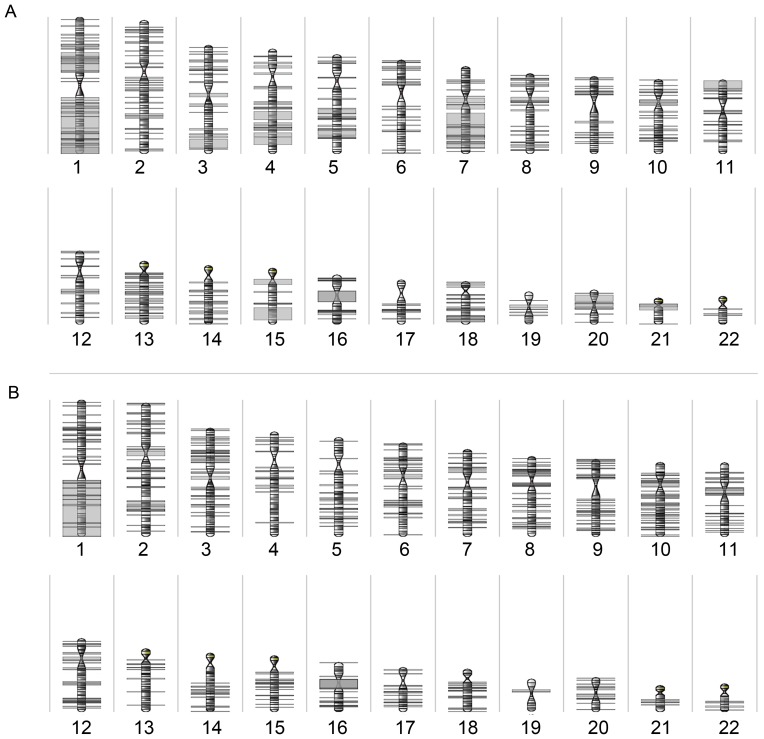
LOH karyogram. Karyogram showing all LOH events detected in (A) the parathyroid carcinomas (T6–T8) and in (B) the parathyroid adenomas (T2–T5) using a 250K SNP array.

LOH was detected on all chromosomes (Figure 5). None of the tumors displayed whole chromosome LOH, however LOH of an entire chromosome arm was detected in three tumors including 1q in T2 and T8, and 7q in T7. Overall, chromosome 1 had the most frequent LOH events, most notably copy number neutral LOH was detected in 1q specifically at 1q25.1 in 5/7 (72%) cases. This region overlaps with several genes for example *RDX6*, *CENPL* and *ANKRD45*. In addition, 4/7 (57%) cases had copy number neutral LOH at 1q21.3 and 1q42.2 overlapping with many genes including *S100A10* and *DISC1*, respectively. The region 1q42.2 largely overlapped with known normal copy number variants. Two tumors (T2 and T8) displayed a large region of copy number neutral LOH of about 18.4 Mb at 1q25.3–32.1 overlapping with the *CDC73* gene. The other five tumors retained heterozygosity at this locus. Similar to the CNAs, the overall LOH events were more frequent among the carcinomas (Figure 5A) compared to the adenomas (Figure 5B).

All the LOH events detected in 2 or more samples together with selected genes are detailed in Table S1.

### Statistical analyses

Statistical analyses regarding differences between adenomas and carcinomas were carried out using Fisher's exact test and power calculations (Table S2). Statistically significant differences between CNAs in adenomas and carcinomas were seen with regards to gross chromosomal losses on 1p (*p* = 0.012), chromosome 13 (*p* = 0.012). Power calculations showed that the total sample size needed for a power of at least 0.80 (p<0.05) generally was around 50 to 100 samples, thereby many of the CNA differences obtained were statistically not significant given the small sample size in this study.

## Discussion

Dysfunction of parafibromin, encoded by the *HRPT2/CDC73* gene, is thought to be a major player in the development of malignant parathyroid tumors [5], [6], [7]. While studies have demonstrated a distinct global gene expression profile of *CDC73* mutated parathyroid tumors compared to *CDC73* wild-type tumors [29], very little is known about the molecular cytogenetic profile. We speculated that mutational inactivation of the *CDC73* gene might lead to distinct chromosomal changes not present in non-mutated cases. Using concurrent genome-wide a-CGH and SNP-array-based LOH analyses, we show that *CDC73-* mutated parathyroid adenomas may have a rather distinctive genetic profile; most strikingly characterized by absence of any significant CNAs on chromosome 1, 11 or 13, while the parathyroid carcinomas displayed similar CNAs to those previously observed in parathyroid carcinomas. This could be due to the recognized high prevalence of *CDC73* gene mutations in the later group. These findings suggest that *CDC73* mutated-parathyroid adenomas may either follow a distinct cytogenetic route apart from carcinomas and *CDC73* wild-type adenomas, or, alternatively, they have not yet developed these changes prior to surgery. Furthermore, even though the sample size in this study was limited, we could see a statistically significant difference regarding loss at chromosomes 1p and 13 in *CDC73* mutated carcinomas as opposed to *CDC73* mutated adenomas. These findings might suggest that genes at these loci might influence the overall malignant behavior in parathyroid tumors with *CDC73* mutations.

All the tumors in this study were selected based on a finding of *CDC73* mutation/s. Apart from tumor T1 and T2, all cases displayed mutations in the coding region with a predicted premature truncation of parafibromin. Tumors T1 and T2 displayed mutations which involved the intronic sequences of *CDC73*. A previously reported mutation in the exon-intron junction has been demonstrated to cause an alternate splicing form of parafibromin by utilizing a cryptic splice donor site further upstream, whereby a protein devoid of codons 35 to 44 of exon 1 is created [8]. Although not demonstrated *per se* for our tumors, it is possible that a similar mechanism is operational in T1, T2 and T5a as their corresponding mutations yield the exact same in-frame deletion as published by Bradley et al [8].

A qPCR-based DNA copy number analysis displayed only one copy of the *CDC73* gene in three of the adenomas (T2, T3 and T4). Interestingly, these three *CDC73*-mutated adenomas have previously been shown to exhibit loss of parafibromin expression using Western blot and immunohistochemistry [22], thereby providing a plausible explanation for the lack of parafibromin in these cases with two separate hits. On the other hand, all the parathyroid carcinomas were diploid or presented with copy number gain for *CDC73*. These results might suggest that parathyroid tumors can develop malignant features even with one functional copy of *CDC73* remaining. However, one should also consider technical issues and contamination with normal tissues as alternative explanations for retained diploid or gain of *CDC73* gene copy detected in these tumors.

Our analyses did not reveal *CDC73* promoter methylation in any of the tumors examined, a finding in line with a previous report [33] suggesting that *CDC73* gene silencing by promoter methylation is not a common mechanism for the gene inactivation.

The overall a-CGH profile of the *CDC73*-mutated tumors revealed partly similar and partly different genetic alterations when compared to previously published studies using conventional CGH [25], [27]. All the three parathyroid carcinomas were characterized by gross chromosomal alterations including gross deletions of 1p, entire chromosome 13 deletions and gain of chromosome 20, while none of them had significant CNAs on chromosome 11. This has also been shown by previous conventional CGH studies in parathyroid carcinomas [25], [27]. Furthermore, many recurrent subchromosomal CNAs were detected which overlapped with several known cancer genes, however further studies of these regions were beyond the scope of this study. Unsupervised whole genome clustering clearly clustered the carcinomas into one group, further confirming the different genetic profile of the carcinomas compared to the non-malignant *CDC73*-mutated adenomas. Therefore, the overall gross chromosomal alterations detected in the carcinoma cases possibly reflect the aggressive clinical behavior and could correlate with the malignant nature of these tumors.

The atypical adenoma (T5b) had an overall cytogenetic profile resembling the carcinomas with the main exception of no changes on chromosome 13. Since many parathyroid tumors have an indolent nature, it is possible to develop malignant transformation many years after the initial diagnosis [34]. However, it is difficult to make any final conclusion based on a single tumor.

In contrast to the carcinomas the adenomas displayed a small extent of CNAs. The most prominent difference to be mentioned is the absence of any significant CNAs on chromosome 1, 11 and 13. Although parathyroid adenomas are known to have frequent losses on chromosomes 1 and 11, losses on chromosome 13 is unknown [25], [26], [28], [35]. This finding supports the theory that *CDC73* gene inactivation possibly directs the tumor towards a different genetic pathway.

The question arises whether the cytogenetic profile differs between cases with hereditary and sporadic diseases. However, when analyzing the CNAs most commonly seen (Table 2), no obvious differences could be perceived when comparing cases T1, T3 and T5 to sporadic cases with somatic mutations (T2, T4, T6–T8). Additionally, since all hereditary cases were adenomas, whereas all carcinomas displayed somatic aberrations, there is also the possibility of an unwanted confounder effect when comparing CNAs from these two groups.

Using SNP array, we could detect LOH events in all the tumors and throughout the genome. Overall, LOH was most frequent in the carcinomas compared to the adenomas. Interestingly, chromosome 1 was the most commonly affected and entire 1q LOH was detected in the two tumors (T2 and T8). This finding implies the possibility of one or more tumor suppressor gene/genes located on 1q arm with implications for parathyroid tumor growth. Since this is the first study of genome-wide LOH analysis in parathyroid tumors, it is not possible to make any comparisons with previously published data. Concerning LOH at specific loci, only two tumors, T2 and T8 revealed LOH at the *CDC73* locus, in which T2 also displayed copy number loss by TaqMan copy number analysis. Furthermore, case T1 only displayed a single chromatogram curve from sequencing analyses, suggesting that a LOH event could be present also here. Using TaqMan copy number assay case T1 was shown to be diploid, indicating that an eventual LOH is copy-neutral. Previous studies using conventional CGH and LOH studies reported frequent losses on chromosome 1p in parathyroid tumors regardless to the *CDC73* status and for which no candidate genes have been found [36], [37].

The main limitation of this study is the small amount of cases employed. *CDC73* gene mutations are exceedingly rare in sporadic parathyroid adenomas, but are more commonly found in cases of sporadic parathyroid carcinomas and in parathyroid tumors from patients with the HPT-JT syndrome, both infrequent conditions. As of this, tumor material with CDC73 mutations is hard to obtain. Further studies in larger cohorts are highly necessary to verify our preliminary results obtained here.

Another weakness in this study is the fact that no *CDC73* wild-type adenomas were included for a-CGH analyses. As of this, our conclusions regarding *CDC73* mutated adenomas and their partly unique cytogenetic profile as compared to *CDC73* wild-type cases are based on the comparison between our current findings and previously published data from this group and others.

In conclusion, we show that *CDC73* -mutated parathyroid adenomas may carry distinct cytogenetic alterations partly different from carcinomas and unselected parathyroid adenomas (with an expected very low frequency of *CDC73* mutations), most noticeably distinguished by the lack of CNAs on chromosome 1, 11 or 13. In addition, *CDC73*-mutated carcinomas display loss at 1p and 13 which is not seen in *CDC73*-mutated adenomas, perhaps providing clues to loci involved in malignant transformation. Additional studies using larger cohorts could potentially address these propositions.

## Supporting Information

Table S1 **LOH raw data generated from the SNP microarray analysis showing LOH events detected in two or more of the tumors analyzed (T2–T8).**
(XLS)

Table S2 **Statistical analysis showing differences in the CNAs detected between the parathyroid adenomas and the carcinomas and the required sample size to detect a statistically significant difference between the two groups of tumors.**
(XLSX)
